# Physical Inactivity, Sedentary Behavior and Quality of Life in the Chilean Population: ENCAVI Results, 2015–2016

**DOI:** 10.3390/healthcare11071020

**Published:** 2023-04-03

**Authors:** Carlos Gonzalez-Torres, Tuillang Yuing, Francisco Berral-de la Rosa, Pablo A. Lizana

**Affiliations:** 1 Laboratory of Epidemiology and Morphological Sciences, Instituto de Biología, Pontificia Universidad Católica de Valparaíso, Valparaíso 2373223, Chile; 2 Programa de Magister en Ciencias Biológicas, Instituto de Biología, Pontificia Universidad Católica de Valparaíso, Valparaíso 2373223, Chile; 3 Facultad de Salud, Escuela de Kinesiología, Universidad Santo Tomás, Viña del Mar 2520000, Chile; 4 Programa de Doctorado en Ciencias de la Actividad Física y del Deporte, Universidad Pablo de Olavide de Sevilla, 41013 Sevilla, Spain; 5 Grupo de Investigación CTS-595, Universidad Pablo de Olavide de Sevilla, 41013 Sevilla, Spain

**Keywords:** physical activity, physical inactivity, sedentary behavior, quality of life

## Abstract

Regular physical activity (PA) is indicated to be one of the main healthy habits that allow populations to achieve a good quality of life (QoL); however, levels of physical inactivity (PI) and sedentary behavior (SB) have risen worldwide, with negative health effects. The aim of this study is to analyze PI and SB levels, as well as their association with QoL in the Chilean population. A secondary analysis was performed based on the database from the 2015–2016 National Quality of Life Survey (ENCAVI) in Chile, using the modules for sociodemographic characteristics, health, and PA. Contingency tables and logistic regressions were conducted to determine the association between PI (low-intensity PA, LIPA; low–moderate PA, LMPA), SB (P75), and QoL adjusted for sociodemographic variables. Approximately 84%, 83%, and 47% of the participants presented LIPA, LMPA, and moderate–high SB, respectively. Participants that presented high PI and SB had lower QoL scores than those who were more active (*p* < 0.05). We observe that, in people with PI (LIPA), there is a higher risk of a low QoL regarding mental and physical components (OR 1.941; OR 1.189, *p* < 0.001) among females (OR 1.473; OR 1.513, *p* < 0.001) and those of a low educational level (OR 2.170; OR 1.410 *p* < 0.001). People with PI (LMPA) increased their risk for a low QoL in regard to mental and physical components (OR 1.750; OR 1.458, *p* < 0.001) among females (OR 1.528; OR 1.507, *p* < 0.001) and those of a low educational level (OR 2.195; OR 1.402 *p* < 0.001). We observe that people with SB (P75) increased their risk of a low QoL concerning physical and mental components (OR 1.475; OR 1.257, *p* < 0.001) for those of the female gender (OR 1.615; OR 1.563, *p* < 0.001) and a low educational level (OR 2.248; OR 1.423 *p* < 0.001). High levels of PI in both intensities and SB impact QoL in the Chilean population. It is crucial to generate public policies for more PA, especially for females and those of lower educational levels.

## 1. Introduction

There is a significant amount of background information describing how one of the crucial healthy habits contributing to a better quality of life (QoL) is performing regular physical activity (PA) [1,2,3]. This is because its benefits lie not only in physical aspects but also in mental and social aspects [4,5]. Even so, recent reports by various researchers have shown that 27.5% of the global population is physically inactive or sedentary. In Latin American and Caribbean countries, this increases to 39.1% [6,7,8]. Inactive lifestyles have shown negative effects on the possibility of integral development, due to facilitating rises in cardiovascular diseases, obesity, diabetes, and cancer, as well as even increasing symptoms associated with mental disorders [9,10,11,12,13].

Sedentary behavior (SB), in contrast to physical inactivity (PI), does involve performing some types of long activities. In such activities, the METs (metabolic equivalents), which are the measurement units for the metabolic index that indicate the energy consumed by an individual and are often used to measure the intensity of exercise, fall under 1.5 METs for sedentary activities; this is a low value when contrasted with light activities of 2.0 METs, such as slowly walking inside the house [14]. This is because the most common activities for a sedentary person are sitting or reclining for long periods of time without interruptions [15]. SB was long considered to be the same as PI, but recent studies have shown that a person can be physically active but still maintain SB, which will bring negative effects for their health, regardless of how active they may be [15,16]. This is because the risk of suffering chronic non-contagious diseases doubles when a person does not incorporate regular exercise to interrupt the long periods of calm that characterize SB [17].

Once the benefits of PA became crucial points to be promoted by the World Health Organization (WHO), research began to focus on SB and PI due to their high levels of associated mortality [18]. In 2010 it was estimated that 3.2 million people died annually due to a lack of PA, placing it as the fourth largest mortality factor in the population [19]. These data are still concerning today, since the COVID-19 pandemic saw reports of major rises in PI and SB levels worldwide due to mandatory lockdowns, teleworking in some work groups [20,21,22], and the impossibility of performing activities outside the home, such as social interactions or open-air sports [23,24].

This makes it important to have control over PI and SB in populations in order to promote good health, since sedentary activities can be present in entire populations. Many of these are imposed due to being associated with types of employment, making it an important challenge to change or eliminate these [16,25]. In this sense, the negative effects of SB can even be incapacitating due to generating different cardiovascular, metabolic, and mental problems, which can even degrade human cognitive functions [26].

In Latin America, periodic national health surveys have made it possible to gain more knowledge about the general health of populations within their respective countries. Most of these surveys have incorporated questions about PA and SB, with the aim of knowing how active their population is [27]. The data have shown that around 40% of Latin American adults, mostly women, do not meet the minimum 150 min of PA recommended by the WHO, and over half spend more than 4 h per day sitting [28]. The main problem with these surveys is the lack of analyses of the data on PI and SB in relation to QoL, which are essential for understanding how inactive the population was prior to the COVID-19 pandemic. This understanding can identify factors that contribute to the acquisition of unhealthy habits that influence populations’ health.

The objective of this study is therefore to analyze PI and SB levels in the Chilean population based on data from the National Quality of Life Survey (ENCAVI) 2015–2016 performed in Chile, in order to learn how an inactive life influences QoL. In this context, it is hypothesized that more inactive and sedentary people will present a lower QoL compared to those who perform regular PA.

## 2. Materials and Methods

A secondary analysis was conducted on the database for the ENCAVI survey in the years 2015–2016, carried out by the Chilean Health Ministry. The ENCAVI aimed to collect information on the living conditions and wellbeing of the population across multiple aspects and included data from 7041 people aged 15 and older from urban and rural zones across the 15 regions of Chile [29].

The ENCAVI 2015–2016 is comprised of 10 modules that include different questions about sociodemographic information, age and indigenous self-identification, health and labor data, QoL, environment and citizen participation, illnesses and accidents, habits and social support, sexuality, education and occupation of household members, and housing conditions.

### 2.1. Participants

**Figure 1:**
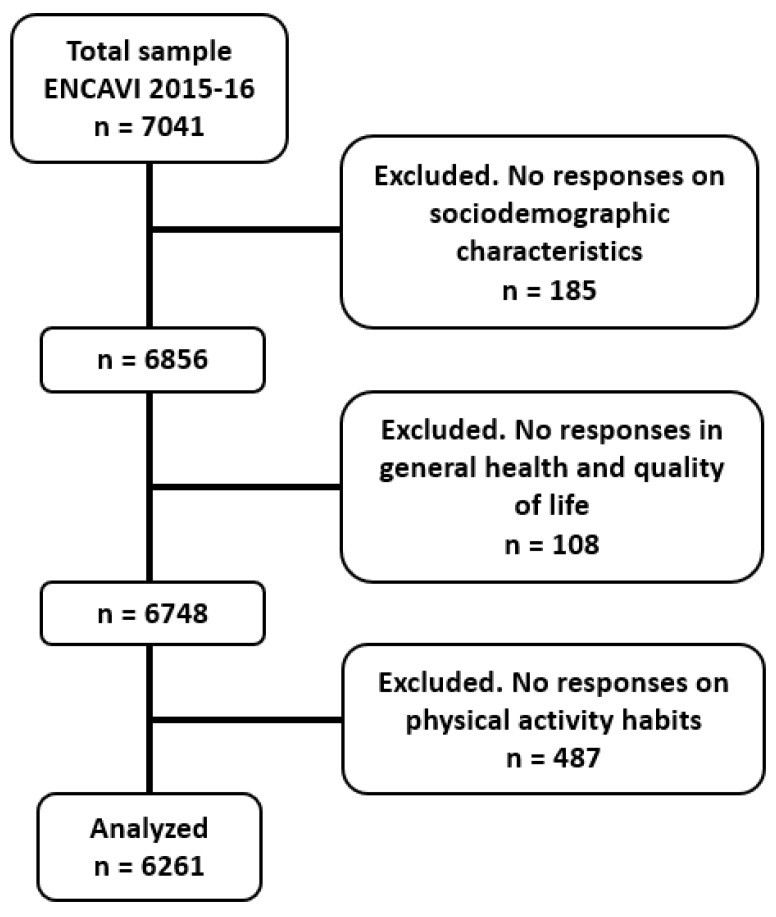
Participants’ flow to achieve the sample size (*n* = 6261).

Out of a total sample of 7041 Chileans included in the ENCAVI 2015–2016, we worked with the following sociodemographic characteristics: gender, age, marital status, educational level, and residential area (urban–rural). Figure 1 describes participants’ flow to achieve the analyzed sample size.

Age was categorized into three groups (≤44, 45–59, and ≥ 60 years), initially based on the categorization from the Chilean National Health Survey of < 45 and ≥ 45 years [30], but adding the age group of ≥ 60 years, as a function of covering different life cycle stages. Educational levels were grouped into three categories (low—up to 8 years, medium—up to 12 years, and high—over 13 years), as was marital status (single, partnered/married, and divorced/widowed) [31,32].

### 2.2. Selection of Variables

To analyze the levels of PI and SB in the Chilean population and its relationship with QoL, we worked with the questions from module I: Interviewee characterization, III: Perceived wellbeing and health, and module VI: Habits [29]. 

As a dependent variable, we chose the interviewees’ QoL contained in module III: Perceived wellbeing and health. This was measured by using questionnaire SF-12, previously validated for the Chilean population [33]. SF-12 groups two QoL summary components: the physical component (PCS) and the mental component (MCS). Each QoL component was scored on a scale of 0 to 100, with 100 being the best possible score and 0 being the worst. The score values were then calculated as standardized values. A score of 50 was used as a cutoff, with scores above 50 indicating a good QoL and scores below 50 indicating a bad QoL. Regarding the reliability of the instrument, it is reported that, for SF-12, the Cronbach’s alpha coefficient oscillates with a mean of 0.67 [34].

For independent variables we selected PI and SB, which were contained in module VI via three different questions: (a) During the last 7 days, on how many days did you perform intense physical activities, including lifting heavy things, digging, aerobic exercise or high-speed bike riding? (Days and hours); (b) During the last 7 days, on how many days did you perform moderate physical activities including moving light weights, regular-speed bike riding, or playing doubles tennis? Do not include walking (Days and hours); and (c) During the last 7 days, how much time did you spend sitting during a work day? Hours per day.

To classify people as physically active or inactive, the replies to questions (a) and (b), which covered performing PA at two intensity levels, were categorized according to the fulfillment of the WHO parameters of at least three days of intensive PA per week and at least five days of moderate PA per week [35,36,37], thereby splitting those who fit into these parameters from those who did not. The latter category was the physically inactive category (low physical activity intensity, LPAI; low–moderate physical activity, LPAM). The previous PI categories were also used for the minimum number of weekly minutes recommended by the WHO, amounting to at least 75 min of intense PA and at least 150 min of moderate PA [35], with the goal of analyzing whether the people who said that they were not physically inactive according to days dedicated to PA were also that way when analyzing their weekly minutes. Finally, question (c), which considered SB in hours per day, was categorized as low (0–3 h), moderate (4–6 h), and high (≥7 h) SB [38,39]. This categorization was obtained based on research with samples of similar characteristics. To evaluate people with greater SB, we also analyzed the P75 of SB, which was set at ≥6 h.

### 2.3. Statistical Analyses

The data were analyzed by using STATA v16 software for Windows (StataCorp. 2019. Stata Statistical Software: Release 16. College Station, TX: StataCorp LLC.). To analyze associations between categorical variables, we used the chi-squared test. In contrast, we applied Wilcoxon’s test for comparisons between continuous variables, given the nonparametric nature of the variables according to the Shapiro–Wilk test. Furthermore, to compare the QoL medians across groups, we applied the Kruskal–Wallis test, Dunn’s post hoc test, and Wilcoxon’s test according to the number of groups examined. All of these statistical tests were conducted for the construction of contingency tables. Finally, six logistic regression models were constructed, maintaining the dependent variable of the cutoff score of 50 of the physical component and mental component of QoL, while for independent variables we used PI (LPAI-LPAM) and the SB P75. All of the logistic regression models were adjusted for sociodemographic characteristics: gender, age, residential area, and educational level. To evaluate the models’ goodness of fit we applied the Hosmer–Lemeshow test, where values over 0.05 for each model indicated that they fit the data.

The results were described via frequencies with percentages (*n*, %) for categorical variables, including PI and SB levels, according to sociodemographic characteristics. In terms of the relationship between PI and SB with QoL values, these were described via their mean and standard deviation (M ± SD), while for the results of the logistic regressions we used their odds ratios (ORs) and confidence intervals. The significance alpha was set at 0.05.

## 3. Results

### 3.1. Sociodemographic Characteristics

The sociodemographic characteristics of the ENCAVI 2015–2016 participants, by gender, appear in Table 1. We can see how, in a total sample of 6261 interviewees, 2351 were men (37.6%) and 3910 were women (63.4%). Eighty percent of the respondents lived in urban zones. For QoL by gender, men presented higher scores compared to women (*p* < 0.001).

### 3.2. PI and SB

Table 2 describes participants as physically inactive or active according to their compliance with the WHO parameters for performing intense PA for at least three days per week. Around 83% of the participants were physically inactive. Furthermore, all of the sociodemographic characteristics had significant differences (*p* < 0.001). The non-fulfillment of intense PA was more frequent for women, urban residents, and people with low as well as medium educational levels. We can also observe that people who did not meet the WHO parameters had lower scores in terms of the PCS and MCS, falling below 50 for the physical component (PCS) (*p* < 0.001).

Table 3 classifies participants as physically inactive or active according to their fulfillment of the WHO parameters for moderate PA at least five days per week, along with the parameters for intense PA. We can see that around 84% of participants were physically inactive and did not meet the minimum WHO requirements, where this is significantly higher in all sociodemographic characteristics. We can also see that people who spent less than five days per week on moderate PA had the lowest scores in terms of QoL PCS and MCS, being under 50 for the PCS (*p* < 0.001).

Table 4 presents the number of minutes for intense and moderate PA among people who said they met the WHO requirements for days doing PA. In terms of the gender variable (*p* = 0.005 and *p* < 0.001), men presented more compliance with the parameters for both intensities, which also occurred with age (*p* = 0.006 and *p* = 0.005), where younger people are the ones who mainly met the WHO parameters. For QoL, we can see that, for both PA intensities, there were significant differences between the PCS and MCS, according to compliance with the moderate and intense PA parameters.

Table 5 presents SB for the population by sociodemographic characteristics. We can observe how around 47% of the sample had moderate–high SB, with women appearing more often in the three SB categories than men did (*p* = 0.002). Something similar occurred with residential area (*p* < 0.001), where people in urban zones had a frequency of over 80% in the three categories compared with those that were rural. 

For the QoL components, we can observe that, in the three SB categories, the PCS did not exceed 50 points and was lower in those with a high SB (*p* = 0.027), while in the MCS the three categories’ scores were ≥50. 

Finally, Table 6 presents the results of the logistic regression between the physical and mental health components, with PI and SB adjusted by sociodemographic characteristics. For LPAI, we can observe how insufficient performance of this type is a risk factor for physical and mental health (OR: 1.941, *p* < 0.001 and OR: 1.189, *p* = 0.024). The same occurs with LPAM, where a lack of performance also presents a risk factor for mental and physical health (OR: 1.750, *p* < 0.001 and OR: 1.568 and OR < 0.001). For SB in high levels, using the P75 of our data as a cutoff, this also presents a risk factor for mental and physical health (OR: 1.475, *p* < 0.001 and OR: 1.257, *p* = 0.001). The variables corresponding to gender and educational level were also associated with lower mental and physical health, while being under 44 and being married or having a partner represented protective factors.

## 4. Discussion

This study analyzed how PI and SB influenced QoL for the Chilean population. We can observe that PI in both intensities and SB are strongly associated with participants’ sociodemographic characteristics. We can report that women, people of lower educational levels, and urban residents present inactive lifestyles. When analyzing variations in physical and mental health according to the PI LIPA, PI LMPA, and SB reports, we observe that the more inactive and sedentary a person is the lower their perceived QoL is. PI in both intensities, high SB, being female, and being of a low educational level were risk factors favoring a poor QoL, while being married/partnered was a health protection factor.

### 4.1. PI and SB Levels

In relation to the SB and PI (in both intensities) patterns studied, we observed a strong association of PI and SB with the participants’ sociodemographic characteristics, such as gender and age (Table 2 and Table 3), which often appear in studies from neighboring countries such as Argentina, Brazil, and Ecuador. Gender, age, and educational level generate significant variations in performing PA [27,28]. In Chile, based on data from the 2016–2017 National Health Survey [30], women from higher age groups were described as having greater PI and performing sedentary activities as compared to younger men. In turn, these PI and SB levels were higher than those reported in 2009–2010 [40,41].

We also observed that high PI LIPA, PI LMPA, and SB levels were present among up to 80% of people living in Chilean urban areas (Table 2, Table 3, Table 4 and Table 5). In this sense, reports from Eurobarometer surveys in Europe that were carried out from 2002 to 2017 indicate that the association between an inactive life and residential area showed that rural areas were the most inactive [42]. In Brazil and the USA, by contrast, performing PA during work was more often reported in rural contexts while recreational PA was more common among people in urban areas, along with the SB measured, with behaviors such as watching TV for more than 3 h daily [43,44]. Despite similar results coming from other countries about inactivity in urban zones, these were not as high as the findings in this study. It is important to note, however, that the comparison studies may have used different instruments and cutoff points for measuring PI and SB. Nevertheless, these comparisons still provide a general understanding of inactive lifestyles in urban areas across different countries. 

One possible explanation for these data is that increased urbanization and the generation of spaces for recreational activities are an important contribution to increased PA indices [45,46]. This could explain the high PI and SB levels in urban areas whose urbanization process did not consider including places to play sports and have social activities, or which do not incentivize regular PA [47,48].

Since 2013, Chile has had a state-run model for developing and creating health habits and lifestyles under the name “Elige Vivir Sano”(“Choose to live healthy”). The basis of this model mentions the lack of public spaces in which to perform PA, which has not changed at this date. This makes it necessary to encourage regular PA in the home [49]. The basis of “Elige Vivir Sano” is coherent with urban zones’ effect and their differences in the PA and QoL patterns of the population, where recent studies have reported that outdoor activities have more psychological benefits for the population when performed in natural areas rather than urban spaces [47]. In turn, the mere presence of significant tree cover and green areas or parks in cities would imply better mental health in the population [50]. This means that the structure of the places where people live can be either a barrier or a facilitator for acquiring healthy habits and a good QoL.

### 4.2. Quality of Life

According to the days dedicated to moderate and intense PA, we can observe that the scores for the mental and physical QoL components are significantly higher among more active people. This difference is greater in the PCS, where inactive people who do not meet the WHO parameters perceived poor physical health. The scientific literature presents an important background that upholds the relationship between a physically active life and good QoL [1,2,3,4,51], stating that performing regular PA is a key habit for people to achieve better mental and physical health perceptions. On the other hand, PI and its harmful effects on QoL, which in the results are observed as results below 50 in the PCS component, also have notable bibliographical support [9,16,52], highlighting that the main effects of a physically inactive lifestyle include greater odds of suffering from chronic non-contagious diseases and a higher risk for at least six different types of cancer, which combine to increase the risk of premature death [12,13]. 

Upon considering the analysis between the PCS and MCS of QoL according to the number of minutes performing intense and moderate PA among people who said they carried out regular PA, this showed that even people who were below the recommended WHO minimum kept their QoL scores over 50. This could indicate that PA is always beneficial, even if done for a few minutes but on a regular basis [53]. Together with this point, recent reports state that at least 20 min per day of moderate to intense PA for 5 days per week can lead to a 16 to 40% reduction in the risk of suffering cardiovascular diseases among adults [54]. This amount of time is similar to the figure studied in this investigation. These points are crucial when we observe that the levels of PI LIPA, PI LMPA, and SB in our sample are quite high within urban zones and lower educational levels. Along with a possible lack of recreational spaces, the high levels of PI LAPI, PI LMPA, and SB in Chile may also be attributed to a lack of awareness about the importance of physical activity, due to limited access to physical education promotion during the early school years [55,56]. In this sense, public policies aiming at promoting overall health and healthy habits should focus on generating as well as restoring open spaces for sports and providing accurate information on the benefits of PA, including strategies to achieve them in shorter time periods.

When analyzing the risk represented by a physically inactive lifestyle for QoL among the studied sample, we can see how low weekly time dedicated to intense PA is a risk factor for physical health (OR: 1.9) and mental health (OR: 1.2). The same is true when dedicating low amounts of weekly time to moderate PA, which also appears as a risk factor for physical health (OR: 1.8) and mental health (OR: 1.5). These results show that both moderate and intense PA are important for maintaining a good QoL, given that having both in balance and with considerable weekly dedication allow for acquiring all of the benefits for good health [57,58]. SB also presents a risk factor for physical health (OR: 1.5) and mental health (OR: 1.3). High SB is hard to control, since regardless of how much PA is performed a person can still have to maintain imposed sedentary activities [15,16], which increase the risk of suffering chronic non-transmissible diseases [17] and can even degrade human cognitive functions [26].

In turn, within the logistic regression models, a low educational level appears as the strongest risk factor for physical health (OR: 2.1) and mental health (OR: 1.4). This has been reported in some studies that showed evidence that as educational levels rose for individuals their QoL rose as well, given that the personal development that can be accessed in various countries depends directly on the level of education achieved [59]. On the other hand, being married or having a partner appears as a protective factor for physical health (OR: 0.8) and mental health (OR: 0.8) while belonging to the first age group of ≤44 years is a protective factor for physical health (OR: 0.3). This may mainly be due to a spouse or partner representing a major support point for the people included in the sample, meaning that negative feelings that directly affect QoL for a single or widowed person, such as loneliness, abandonment, and mourning, were not present in these people in the same way [60,61]. Younger people in general are also described as being more physically active, given that other age groups have already begun to present the physiological changes related to aging that affect both their nervous systems and musculoskeletal systems, directly impacting their general health and sporting abilities [62,63].

The results obtained in this study, which show high levels of PI LIPA, PI LMPA, and SB among the Chilean population that participated in the ENCAVI 2015–2016, align with previous studies in Chile that observed that incidences of physically inactive lifestyles have increased with each national health survey carried out [27,28]. It is notable that these data represent a society before the COVID-19 pandemic, an event which generated major changes in habits among the global population [20,22,24]. Linking this high PI in terms of both intensities and SB behaviors with QoL generates more of a background with which to go into further depth about some of the factors discussed in this study which may favor inactive lifestyles in our population. These include a lack of public spaces for doing open-air sports or a lack of awareness about the benefits of exercise that may exist among people with low educational levels.

### 4.3. Limitations

The main limitation of this study comes from the cross-sectional nature of the database. This allows us to obtain an information snapshot from participants, rather than permitting data evolution analyses. Another limitation is that adolescents and adults are treated as individuals aged ≤45, which may not accurately reflect the age-related differences in the variables of interest. Nevertheless, the strong national representativeness of the ENCAVI 2015–2016, arising from the number of participants it drew from, nationwide, is a core strength of the study, since it allows us to approach the national reality on a large scale.

## 5. Conclusions

The secondary analysis conducted on the ENCAVI 2015–2016 showed high PI and SB levels in the Chilean population, mainly among females, urban residents, and people of low educational levels. Overall, these represented major risk factors for QoL in the studied sample, mainly reflected in scores below 50 for the PCS. Even so, the participants who performed regular PA, even for a few minutes per day, had a satisfactory QoL. These results should drive public policies that favor a more active lifestyle for the Chilean population.

## Figures and Tables

**Table 1:** Sociodemographic characteristics and quality of life by gender (*n* = 6261).

		Total Sample (*n* = 6261)	Male (*n* = 2351)	Female (*n* = 3910)	*p*
Age ^c^		47.18 ± 18.60	47.09 ± 19.26	47.46 ± 18.29	0.623 ^a^
≤44		2843 (45.41)	1055 (44.87)	1788 (45.73)	0.752 ^b^
45–59		1612 (25.75)	606 (25.78)	1006 (25.72)	
≥60		1806 (28.85)	690 (29.35)	1116 (28.54)	
Marital Status					<0.001 ^b^
Single		1593 (25.40)	564 (24.00)	1029 (26.30)	
Married/partnered		3763 (60.10)	1486 (63.20)	2277 (58.20)	
DWW		905 (14.50)	301 (12.80)	604 (15.04)	
Residence Area					0.001 ^b^
Urban		5209 (83.20)	2004 (85.20)	3205 (82.00)	
Rural		1052 (16.80)	347 (14.80)	705 (18.00)	
Educational Level					<0.001 ^b^
Low		2395 (38.30)	835 (35.50)	1560 (39.90)	
Medium		2521 (40.30)	959 (40.80)	1562 (39.90)	
High		1345 (21.50)	237 (23.70)	788 (20.20)	
Quality of Life ^c^					<0.001 ^a^
PCS		49.14 ± 9.58	50.41 ± 8.73	48.27 ± 10.09	
MCS		51.65 ± 8.97	53.21 ± 7.99	51.03 ± 9.43	

^a^ Wilcoxon’s test; ^b^ Chi-squared; ^c^ data expressed as mean and standard deviation; DWW: divorced widow widower; PCS: physical component summary; and MCS: mental component summary.

**Table 2:** Comparison of compliance with the WHO-designated parameters for intense PA (days) according to sociodemographic characteristics.

	WHO Parameters for Intense PA		
	Non-Compliant (<3 Days)	Compliant (≥3 Days)	*p*
	Inactive Person	Active Person	
Total sample	5236 (83.63)	1025 (16.37)	
Gender			<0.001 ^b^
Male	1765 (33.70)	586 (57.20)	
Female	3471 (66.30)	439 (42.80)	
Age			<0.001 ^b^
≤44	2216 (42.32)	627 (61.17)	
45–59	1332 (25.44)	280 (27.32)	
≥60	1688 (32.24)	118 (11.51)	
Marital Status			0.001 ^b^
Single	1310 (25.00)	283 (27.60)	
Married/partnered	3119 (59.60)	644 (62.80)	
DWW	807 (15.40)	98 (9.60)	
Residence Area			<0.001 ^b^
Urban	4311 (82.30)	898 (87.60)	
Rural	925 (17.70)	127 (12.40)	
Educational Level			<0.001 ^b^
Low	2144 (40.90)	251 (24.50)	
Medium	2015 (38.50)	506 (49.40)	
High	1077 (20.60)	268 (26.10)	
Quality of Life ^c^			<0.001 ^a^
PCS	48.35 ± 10.02	52.79 ± 6.37	
MCS	51.56 ± 9.19	53.29 ± 7.64	

^a^ Wilcoxon’s test; ^b^ Chi-squared; ^c^ data expressed as mean and standard deviation; DWW: divorced widow widower; PCS: physical component summary; and MCS: mental component summary.

**Table 3:** Comparison of compliance with the WHO-designated parameters for moderate PA (days) according to sociodemographic characteristics.

		WHO Parameters for Moderate PA		
		Non-Compliant (<5 days)	Compliant (≥5 days)	*p*
		Inactive Person	Active Person	
Total sample		5220 (83.37)	1041 (16.63)	
Gender				<0.001 ^b^
Male		1842 (35.30)	509 (48.90)	
Female		3378 (64.70)	532 (51.10)	
Age				<0.001 ^b^
≤44		2260 (43.30)	583 (56.00)	
45–59		1336 (25.59)	276 (26.51)	
≥60		1624 (31.11)	182 (17.48)	
Marital Status				<0.001 ^b^
Single		1338 (25.60)	255 (24.50)	
Married/partnered		3070 (58.80)	693 (66.60)	
DWW		812 (15.60)	93 (8.90)	
Residence Area				0.030 ^b^
Urban		4319 (82.70)	890 (85.50)	
Rural		901 (17.30)	151 (14.50)	
Educational Level				<0.001 ^b^
Low		2104 (40.30)	291 (28.00)	
Medium		2035 (39.00)	486 (46.70)	
High		1081 (20.70)	264 (25.40)	
Quality of Life ^c^				<0.001 ^a^
PCS		48.49 ± 9.96	51.99 ± 7.29	
MCS		51.45 ± 9.10	53.85 ± 8.04	

^a^ Wilcoxon’s test; ^b^ Chi-squared; ^c^ data expressed as mean and standard deviation; DWW: divorced widow widower; PCS: physical component summary; and MCS: mental component summary.

**Table 4:** Comparison of compliance with the WHO-designated parameters for intense and moderate PA (minutes) according to sociodemographic characteristics.

	WHO Parameters for Intense PA (*n* = 1539)			WHO Parameters for Moderate PA (*n* = 2335)		
	Non-Compliant (<75 min)	Compliant (≥75 min)	*p*	Non-Compliant (<150 min)	Compliant (≥150 min)	*p*
	Inactive Person	Active Person		Inactive Person	Active Person	
Gender			0.005 ^b^			<0.001 ^b^
Male	144 (45.00)	655 (53.73)		879 (44.94)	208 (54.88)	
Female	176 (55.00)	564 (46.27)		1077 (55.06)	171 (45.12)	
Age			0.006 ^b^			0.005 ^b^
≤44	173 (54.06)	775 (63.58)		1170 (59.82)	194 (51.19)	
45–59	97 (30.31)	306 (25.10)		472 (24.13)	117 (30.87)	
≥60	50 (15.63)	138 (11.32)		314 (16.05)	68 (17.94)	
Marital Status			0.087 ^b^			0.411 ^b^
Single	69 (21.56)	337 (27.65)		476 (24.34)	100 (26.39)	
Married/partnered	214 (66.88)	757 (62.10)		1276 (65.24)	247 (65.17)	
DWW	37 (11.56)	125 (10.25)		204 (10.43)	32 (8.44)	
Residence Area			0.327 ^b^			0.001 ^b^
Urban	278 (86.88)	1083 (88.84)		1740 (88.96)	314 (82.85)	
Rural	42 (13.13)	136 (11.16)		216 (11.04)	65 (17.15)	
Educational Level			0.785 ^b^			<0.001 ^b^
Low	76 (23.75)	306 (25.10)		476 (24.34)	111 (29.29)	
Medium	153 (47.81)	557 (45.69)		852 (43.56)	212 (55.94)	
High	91 (28.44)	356 (29.20)		628 (32.11)	56 (14.78)	
Quality of Life ^c^						
PCS	51.67 ± 7.14	52.89 ± 6.22	0.004 ^a^	51.35 ± 7.35	52.22 ± 6.75	0.001 ^a^
MCS	52.79 ± 7.92	54.66 ± 7.73	< 0.001 ^a^	52.16 ± 8.93	54.00 ± 7.81	<0.001 ^a^

^a^ Wilcoxon’s test; ^b^ Chi-squared; ^c^ data expressed as mean and standard deviation; DWW: divorced widow widower; PCS: physical component summary; and MCS: mental component summary.

**Table 5:** Sedentary behavior of the population according to sociodemographic characteristics.

	Sedentary Time				
	Low (0–3 Hours) ^1^	Moderate (4–6 Hours) ^2^	High (≥7 Hours) ^3^	*p*	Post Hoc ^c^
Total sample	3319 (53.01)	2232 (35.65)	710 (11.34)		
Gender				0.002 ^a^	
Male	1202 (36.20)	841 (37.70)	308 (43.4)		
Female	2117 (63.80)	1394 (62.30)	402 (56.6)		
Age				<0.001 ^a^	
≤44	1473 (44.38)	1024 (45.88)	346 (48.73)		
45–59	933 (28.11)	508 (22.76)	171 (24.08)		
≥60	913 (27.51)	700 (31.36)	193 (27.18)		
Marital Status				<0.001 ^a^	
Single	837 (25.20)	554 (24.80)	202 (28.50)		
Married/partnered	2070 (62.40)	1320 (59.10)	373 (52.50)		
DWW	412 (12.40)	358 (16.00)	135 (19.00)		
Residence Area				<0.001 ^a^	
Urban	2658 (80.10)	1949 (87.30)	602 (84.80)		
Rural	661 (19.90)	283 (12.70)	108 (15.20)		
Educational Level				<0.001 ^a^	
Low	1304 (39.30)	806 (36.10)	285 (40.10)		
Medium	1379 (41.50)	869 (38.90)	273 (38.50)		
High	636 (19.20)	57 (25.00)	152 (21.40)		
Quality of Life ^d^					
PCS	49.56 ± 8.94	49.07 ± 9.78	46.82 ± 11.93	0.027 ^b^	1 > 3; 2 > 3
MCS	51.82 ± 8.62	52.33 ± 9.23	50.42 ± 9.64	<0.001 ^b^	1 > 3; 2 > 3

^a^ Chi-squared test; ^b^ Kruskal–Wallis test with Dunn’s post hoc test; ^c 1^: low sedentary time; ^2^: moderate sedentary time; and ^3^: high sedentary time; ^d^ data expressed as mean and standard deviation; DWW: divorced widow widower; PCS: physical component summary; and MCS: mental component summary.

**Table 6:** Logistic regressions for the association between the physical and mental health components according to physical inactivity and sedentary behavior, adjusted for sociodemographic characteristics.

		Physical Component Summary		Mental Component Summary	
		Scores < 50		Scores < 50	
		Odds Ratio (95% CI)	*p*	Odds Ratio (95% CI)	*p*
Intense Physical Activity					
<3 days/week		1.941 (1.631–2.311)	<0.001	1.189 (1.023–1.382)	0.024
Gender (female)		1.473 (1.307–1.660)	<0.001	1.513 (1.353–1.692)	<0.001
Age (≤44 years)		0.273 (0.242–0.309)	<0.001	0.972 (0.868–1.089)	0.624
Marital status (Married/partnered)		0.765 (0.682–0.858)	<0.001	0.837 (0.752–0.931)	0.001
Area (urban)		0.921 (0.682–0.858)	0.280	1.022 (0.887–1.178)	0.760
Educational level (<8 years)		2.170 (1.925–2.445)	<0.001	1.410 (1.255–1.584)	<0.001
Hosmer–Lemeshow test ^a^		0.376		0.523	
Moderate Physical Activity					
<5 days/week		1.750 (1.484–2.064)	<0.001	1.458 (1.254–1.694)	<0.001
Gender (female)		1.528 (1.357–1.719)	<0.001	1.507 (1.349–1.684)	<0.001
Age (≤44 years)		0.268 (0.237–0.303)	<0.001	0.978 (0.873–1.095)	0.694
Marital status (Married/partnered)		0.777 (0.693–0.871)	<0.001	0.846 (0.761–0.942)	0.002
Area (urban)		0.912 (0.786–1.059)	0.229	1.020 (0.885–1.176)	0.781
Educational level (<8 years)		2.195 (1.948–2.473)	<0.001	1.402 (1.247–1.575)	<0.001
Hosmer–Lemeshow test ^a^		0.548		0.663	
Sedentary Behavior					
≥6 h/week		1.475 (1.280–1.700)	<0.001	1.257 (1.103–1.433)	0.001
Gender (female)		1.615 (1.435–1.817)	<0.001	1.563 (1.400–1.746)	<0.001
Age (≤44 years)		0.259 (0.229–0.293)	<0.001	0.954 (0.852–1.067)	0.408
Marital status (Married/partnered)		0.777 (0.693–0.872)	<0.001	0.846 (0.760–0.941)	0.002
Area (urban)		0.901 (0.776–1.046)	0.171	1.011 (0.877–1.165)	0.877
Educational level (<8 years)		2.248 (1.996–2.532)	<0.001	1.423 (1.267–1.599)	<0.001
Hosmer–Lemeshow test ^a^		0.624		0.796	

^a^ A value above 0.05 indicates that the model fits the data.

## Data Availability

The datasets generated and/or analyzed during the current study are available from the corresponding author on reasonable request.
